# Supplementary material for: Analysis of PPARγ Signaling Activity in Psoriasis
Source: Int J Mol Sci. 2021 Aug 10;22(16):8603. doi: 10.3390/ijms22168603 (PMC8395241; doi:10.3390/ijms22168603)
Supplement: Supplementary file 1 [file ijms-22-08603-s001.zip › Supplemental materials_Analysis of PPARg signaling activity in psoriasis/Pathway models/Models images and html files/Differentiation of psoriatic T cells/4902.html]

GATA3


# Protein GATA3

|  |  |
| --- | --- |
| URN | urn:agi-llid:2625 |
| Total Entities | 0 |
| Connectivity | 3021 |
| Name | GATA3 |
| Description | GATA binding protein 3 |
| Notes | This gene encodes a protein which belongs to the GATA family of transcription factors. The protein contains two GATA-type zinc fingers and is an important regulator of T-cell development and plays an important role in endothelial cell biology. Defects in this gene are the cause of hypoparathyroidism with sensorineural deafness and renal dysplasia. [provided by RefSeq, Nov 2009] |
| Primary Cell Localization | Nucleus |
| Class | Transcription factor |

---

|  |  |
| --- | --- |
| Pathway | T-Cell Maturation (Hypothesis) |
|  | Th2-Cell Differentiation |
|  | Acute Phase in Atopic Dermatitis |
|  | Atopic Dermatitis |
|  | T-Cells Differentiation Block in Psoriasis |
|  | Proteins with Altered Expression in Psoriasis |
|  | Th2-Cell Function in Systemic Lupus Erythematosus |
|  | Vitiligo |
|  | T-Cell Cytotoxic Response against Melanocytes in Vitiligo |
|  | Immune System Activation in Hyperthyroidism |
|  | Proteins Involved in Hypoparathyroidism |
|  | Hypoparathyroidism, Primary |
|  | CD4+T-Cell Response in Celiac Disease |
|  | B-Cell Activation in Crohn's Disease |
|  | Th2-Cell Function in Ulcerative Colitis |
|  | Hodgkin Lymphoma |
|  | Hodgkin and Reed-Sternberg Cells Reprogramming |
|  | T-Cell Acute Lymphoblastic Leukemia |
|  | Proteins Involved in Pancreatic Cancer |
|  | Th2-Cell Response in Asthma |
|  | Th2-Cells Function in Systemic Scleroderma |
|  | Proteins with Altered Expression in Systemic Scleroderma |
|  | CD4+ T-Cell Function Decline in HIV |
|  | Gluten Impact on Neuronal System (Hypothesis) |
|  | PHOX2B Effects in Neuroblastoma |
|  | Neuroblastoma |
|  | Proteins Involved in Breast Cancer Related to ESR1 Signaling Pathway |
|  | Proteins Involved in Endometriosis |
|  | Proteins Involved in Spontaneous Abortion |
|  | Proteins Involved in Hearing Loss |
|  | IL15 Expression Targets |
|  | CD40 -> Expression Targets in Thymus |
|  | NOTCH -> Expression Targets in Thymus |
|  | NOTCH Receptors -> Receptors Expression Targets in Lung |
|  | TCR -> NF-kB Expression Targets |
|  | TCR -> NFAT Expression Targets |
|  | PPAR Psoriasis |
|  | Model of PPARG signaling in psoriasis |
|  | PPARG negative regulators and targets |
|  | Model of PPARG related pathways in psoriasis (short version) |
|  | 2\_Acute phase initiation in atopic dermatitis |
|  | 1\_Differentiation of psoriatic T cells |
|  | 1\_Th2 cell response in asthma |
|  | 2\_Intestine epithelial cells drive immunological responses in ulcerative colitis |
|  | 4\_1\_Polymorphisms associated with inflammatory bowel diseases |
|  | Differentiation of psoriatic T cells |

---

|  |  |
| --- | --- |
| Group | Genes with Mutations Associated with Hypoparathyroidism |
|  | Genes with Mutations Associated with Breast Cancer |

---

|  |  |
| --- | --- |
| MedScan ID | 2625 |

---

|  |  |
| --- | --- |
| LocusLink ID | 2625 |
|  | 14462 |
|  | 85471 |

---

|  |  |
| --- | --- |
| Alias | OTTHUMP00000019071 |
|  | RP23-276D17.1 |
|  | OTTHUMP00000019070 |
|  | transcription factor GATA3 |
|  | MGC5445 |
|  | Hypoparathyroidism, sensorineural deafness, renal dysplasia gene |
|  | GATA binding protein-3 |
|  | GATA-binding protein 3 |
|  | GATA binding protein 3 |
|  | GATA 3 transcription factor |
|  | GATA-binding protein-3 |
|  | GATA3 |
|  | OTTMUSP00000011932 |
|  | MGC5199 |
|  | GATA-binding factor 3 |
|  | GATA binding protein III |
|  | Trans-acting T-cell specific transcription factor GATA-3 |
|  | MGC2346 |
|  | Trans-acting T-cell specific transcription factor GATA III |
|  | HDR |
|  | HDRS |
|  | trans-acting T-cell-specific transcription factor GATA-3 |
|  | jal |
|  | Gata-3 |

---

|  |  |
| --- | --- |
| GO ID | 0003677 |
|  | 0001228 |
|  | 0003700 |
|  | 0000981 |
|  | 0001227 |
|  | 0070888 |
|  | 0071837 |
|  | 0000978 |
|  | 0001158 |
|  | 0005134 |
|  | 0000987 |
|  | 0003713 |
|  | 0008134 |
|  | 0044212 |
|  | 0000976 |
|  | 0008270 |
|  | 0050852 |
|  | 0045064 |
|  | 0031929 |
|  | 0048646 |
|  | 0009653 |
|  | 0003180 |
|  | 0007411 |
|  | 0007596 |
|  | 0061290 |
|  | 0003215 |
|  | 0001709 |
|  | 0048469 |
|  | 0071773 |
|  | 0035457 |
|  | 0071353 |
|  | 0071356 |
|  | 0006338 |
|  | 0090102 |
|  | 0019221 |
|  | 0006952 |
|  | 0048589 |
|  | 0043583 |
|  | 0035162 |
|  | 0048568 |
|  | 0030218 |
|  | 0006959 |
|  | 0002520 |
|  | 0001701 |
|  | 0045087 |
|  | 0042472 |
|  | 0072643 |
|  | 0072602 |
|  | 0001822 |
|  | 0002088 |
|  | 0072676 |
|  | 0008584 |
|  | 0060374 |
|  | 0060231 |
|  | 0001823 |
|  | 1901536 |
|  | 0045786 |
|  | 2000146 |
|  | 0008285 |
|  | 2000607 |
|  | 2000352 |
|  | 0045599 |
|  | 2000703 |
|  | 2000734 |
|  | 0050728 |
|  | 0032689 |
|  | 0032703 |
|  | 0033600 |
|  | 0000122 |
|  | 0045892 |
|  | 0072179 |
|  | 0072178 |
|  | 0001764 |
|  | 0042421 |
|  | 0071599 |
|  | 0060017 |
|  | 0035898 |
|  | 0060037 |
|  | 0014065 |
|  | 0045582 |
|  | 2000553 |
|  | 0010595 |
|  | 0071442 |
|  | 2000617 |
|  | 2000667 |
|  | 0032753 |
|  | 2000664 |
|  | 0051897 |
|  | 0009967 |
|  | 2000611 |
|  | 0045944 |
|  | 2000679 |
|  | 0045893 |
|  | 0072107 |
|  | 0009791 |
|  | 0002572 |
|  | 0016579 |
|  | 0043370 |
|  | 2000683 |
|  | 0042035 |
|  | 0030856 |
|  | 2000114 |
|  | 1902036 |
|  | 0061085 |
|  | 0051569 |
|  | 0072182 |
|  | 0043523 |
|  | 0010975 |
|  | 0043627 |
|  | 0045471 |
|  | 0010332 |
|  | 0009615 |
|  | 0007165 |
|  | 0048485 |
|  | 0045061 |
|  | 0048538 |
|  | 0006366 |
|  | 0001806 |
|  | 0035799 |
|  | 0072197 |
|  | 0060676 |
|  | 0060065 |
|  | 0003281 |
|  | 0000790 |
|  | 0005654 |
|  | 0005634 |
|  | 0000977 |
|  | 0046872 |
|  | 0043565 |
|  | 0003712 |
|  | 0030217 |
|  | 0033077 |
|  | 0001775 |
|  | 0000902 |
|  | 0071345 |
|  | 0048872 |
|  | 0002376 |
|  | 0010629 |
|  | 0007399 |
|  | 0030182 |
|  | 0045597 |
|  | 0001819 |
|  | 0010628 |
|  | 0032736 |
|  | 0032754 |
|  | 0006357 |
|  | 0006355 |
|  | 0042493 |
|  | 0007507 |
|  | 0072001 |
|  | 0009887 |
|  | 0009888 |
|  | 0045165 |
|  | 0006351 |
|  | 0001077 |
|  | 0001076 |
|  | 0048565 |
|  | 0001078 |
|  | 0001046 |
|  | 0048468 |
|  | 0000979 |
|  | 0001085 |
|  | 0003682 |
|  | 0001071 |
|  | 0005730 |
|  | 0005667 |
|  | 0005623 |
|  | 0005622 |
|  | 0010552 |
|  | 0016563 |
|  | 0043193 |

---

|  |  |
| --- | --- |
| KEGG ID | hsa:2625 |
|  | mmu:14462 |
|  | rno:85471 |

---

|  |  |
| --- | --- |
| Organism | Homo sapiens {Organism urn:agi-taxid:9606} |
|  | Mus musculus {Organism urn:agi-taxid:10090} |
|  | Rattus norvegicus {Organism urn:agi-taxid:10116} |
|  | Mus musculus |
|  | Homo sapiens |
|  | Rattus norvegicus |

---

|  |  |
| --- | --- |
| Mouse chromosome position | 2 6.69 cM |
|  | 2 7.0 cM |

---

|  |  |
| --- | --- |
| OMIM ID | 131320 |
|  | 146255 |

---

|  |  |
| --- | --- |
| Rat chromosome position | 17q12.3 |

---

|  |  |
| --- | --- |
| Hugo ID | 4172 |
|  | HGNC:4172 |

---

|  |  |
| --- | --- |
| Human chromosome position | 10p14 |
|  | 10p15 |

---

|  |  |
| --- | --- |
| Swiss-Prot Accession | P23771 |
|  | P23771.1 |
|  | P23772 |
|  | Q3U0R5 |
|  | P23772.1 |
|  | Q99NH5 |
|  | Q5VWG7 |
|  | Q5VWG8 |
|  | Q96J16 |
|  | Q3TZD6 |
|  | Q3TZS0 |
|  | Q3U223 |

---

|  |  |
| --- | --- |
| PIR ID | A39794 |
|  | B39794 |

---

|  |  |
| --- | --- |
| GenBank ID | NC\_000010 |
|  | XM\_005252443 |
|  | XP\_005252500 |
|  | XM\_005252442 |
|  | XP\_005252499 |
|  | NM\_001002295 |
|  | NP\_001002295 |
|  | NM\_002051 |
|  | NP\_002042 |
|  | NG\_015859 |
|  | AL390294 |
|  | AY497006 |
|  | AAR32096 |
|  | CH471072 |
|  | EAW86367 |
|  | EAW86368 |
|  | FB571433 |
|  | CAR95134 |
|  | FB571435 |
|  | CAR95135 |
|  | HI179114 |
|  | CBX53865 |
|  | HI179116 |
|  | CBX53866 |
|  | JC009146 |
|  | CDL93507 |
|  | KT583933 |
|  | X73519 |
|  | AI024501 |
|  | AI686327 |
|  | AK298213 |
|  | BAG60483 |
|  | BC003070 |
|  | AAH03070 |
|  | BC006793 |
|  | AAH06793 |
|  | BC006839 |
|  | BG287052 |
|  | BM152193 |
|  | M69106 |
|  | AAA35870 |
|  | X55037 |
|  | CAA38877 |
|  | X55122 |
|  | CAA38916 |
|  | X58072 |
|  | CAA41102 |
|  | P23771 |
|  | NC\_000068 |
|  | XM\_006497355 |
|  | XP\_006497418 |
|  | NM\_008091 |
|  | NP\_032117 |
|  | XM\_006497354 |
|  | XP\_006497417 |
|  | NM\_001355110 |
|  | NM\_001355111 |
|  | NM\_001355112 |
|  | AH015706 |
|  | AH015707 |
|  | AL845529 |
|  | AL928704 |
|  | CH466542 |
|  | EDL07996 |
|  | X73520 |
|  | Z33620 |
|  | AA759391 |
|  | AK053728 |
|  | AK090089 |
|  | AK155549 |
|  | BAE33319 |
|  | AK156634 |
|  | BAE33786 |
|  | AK157625 |
|  | BAE34137 |
|  | AK157939 |
|  | BAE34273 |
|  | AK161099 |
|  | BC062915 |
|  | AAH62915 |
|  | X55123 |
|  | CAA38917 |
|  | P23772 |
|  | NC\_005116 |
|  | XM\_017600666 |
|  | XP\_017456155 |
|  | XM\_008771894 |
|  | XP\_008770116 |
|  | XM\_006254253 |
|  | XP\_006254315 |
|  | XM\_017600667 |
|  | XP\_017456156 |
|  | XM\_008771893 |
|  | XP\_008770115 |
|  | NM\_133293 |
|  | NP\_579827 |
|  | AC\_000085 |
|  | AABR07028502 |
|  | AAHX01092527 |
|  | AAHX01092528 |
|  | AAHX01092529 |
|  | CH473990 |
|  | EDL78630 |
|  | AB000217 |
|  | AY024364 |
|  | AAK00586 |
|  | NC\_018921 |
|  | AMYH02022091 |
|  | AM392571 |
|  | CAL37449 |
|  | AM392688 |
|  | CAL37566 |
|  | XM\_006497353 |
|  | XP\_006497416 |
|  | XM\_006497356 |
|  | XP\_006497419 |
|  | AC\_000024 |
|  | AAHY01013856 |
|  | DQ400110 |
|  | ABD66080 |
|  | DQ400111 |
|  | DQ400112 |
|  | DQ400113 |
|  | DQ400115 |
|  | DQ400118 |
|  | ABD66081 |
|  | DQ400119 |
|  | DQ400120 |
|  | DQ400121 |
|  | DQ400123 |
|  | AC\_000142 |
|  | ABBA01034729 |
|  | XM\_006254255 |
|  | XP\_006254317 |
|  | XM\_006254254 |
|  | XP\_006254316 |
|  | AABR06092817 |
|  | AABR06092818 |
|  | CAH73143 |
|  | CAH73144 |
|  | CAM19357 |
|  | CAM19358 |
|  | NT\_008705 |
|  | NW\_924584 |
|  | NW\_001837931 |
|  | AC\_000053 |
|  | NT\_039202 |
|  | NW\_001030686 |
|  | Q3TZD6 |
|  | Q3TZS0 |
|  | Q3U0R5 |
|  | Q3U223 |
|  | NW\_047492 |
|  | NW\_001084730 |
|  | Q99NH5 |

---

|  |  |
| --- | --- |
| Swiss-Prot ID | GATA3\_HUMAN |
|  | GATA3\_MOUSE |
|  | Q3U0R5\_MOUSE |
|  | Q99NH5\_RAT |
|  | Q5VWG7\_HUMAN |
|  | Q5VWG8\_HUMAN |

---

|  |  |
| --- | --- |
| Cell Localization | Nucleus |

---

|  |  |
| --- | --- |
| Ensembl ID | ENSG00000107485 |
|  | ENSP00000368632.3 |
|  | ENST00000379328.9 |
|  | ENSP00000341619.3 |
|  | ENST00000346208.4 |
|  | ENSMUSG00000015619 |
|  | ENSMUSP00000100041.3 |
|  | ENSMUST00000102976.3 |
|  | ENSRNOG00000019336 |
|  | ENSRNOP00000026187.3 |
|  | ENSRNOT00000026187.5 |
|  | ENST00000379328.8 |
|  | ENSP00000368632 |
|  | ENST00000379328 |
|  | ENSP00000341619 |
|  | ENST00000346208 |
|  | ENSMUSP00000100041 |
|  | ENSMUST00000102976 |
|  | ENSRNOP00000026187 |
|  | ENSRNOT00000026187 |

---

|  |  |
| --- | --- |
| MGI ID | MGI:95663 |
|  | 95663 |

---

|  |  |
| --- | --- |
| RGD ID | 621250 |

---

|  |  |
| --- | --- |
| Unigene ID | Hs.524134 |
|  | Mm.313866 |
|  | Rn.229174 |
|  | Rn.92350 |

---

|  |  |
| --- | --- |
| Homologene ID | 1550 |

---

|  |  |
| --- | --- |
| Shape | O-vertex |

---

|  |  |
| --- | --- |
| IPI ID | IPI00012901 |
|  | IPI00216199 |
|  | IPI00135883 |
|  | IPI00198958 |
|  | IPI00756271 |

---
